# Ten years of experience with elbow native joint arthritis: a multicenter retrospective cohort study

**DOI:** 10.5194/jbji-10-25-2025

**Published:** 2025-02-20

**Authors:** Pansachee Damronglerd, Ryan Bijan Khodadadi, Said El Zein, Jack William McHugh, Omar M. Abu Saleh, Mark Edward Morrey, Aaron Joseph Tande, Gina Ann Suh

**Affiliations:** 1 Division of Public Health, Infectious Diseases and Occupational Medicine, Mayo Clinic, 200 First Street SW, Rochester, Minnesota, USA; 2 Division of Infectious Diseases, Faculty of Medicine, Thammasat University, Pathum Thani, Thailand; 3 Department of Orthopedic Surgery, Mayo Clinic, Rochester, Minnesota, USA

## Abstract

**Background**: Elbow native joint septic arthritis (NJSA) is a rare condition, constituting 6 %–9 % of all native septic arthritis cases. It is associated with elevated mortality and morbidity. This study aims to clarify the characteristics, management, and outcomes of elbow NJSA. **Methods**: We retrospectively analyzed adults diagnosed with elbow NJSA who underwent surgical intervention at Mayo Clinic facilities from January 2012 to December 2021. Diagnosis relied on clinical presentation, synovial fluid white blood cell (WBC) count, and aspiration or operative cultures. **Results**: Among 557 patients with NJSA during the study time frame, 19 (3.4 %) were found to have elbow NJSA. The median age of these patients was 64 years. Joint aspirations were conducted in 16 cases (84.2 %). The median synovial fluid WBC count was 43 139 cells mm^−3^. Crystals were observed in three patients (15.8 %). Synovial fluid and operative tissue samples revealed 12.5 % and 20 % positive Gram stains, mostly indicating Gram-positive cocci clusters. Open arthrotomy (72.2 %) was the predominant surgical approach, and three patients (16.7 %) required reoperation within 90 d. The median antimicrobial therapy duration was 30 d (interquartile range: 22–44 d). Non-tuberculosis mycobacterium (NTM) was detected in two patients, with a treatment duration of 274 and 374 d, respectively. Complications included joint contracture and joint resection. **Conclusions**: Elbow NJSA is an infrequent condition associated with significant complications, such as the necessity for reoperation. Although the synovial fluid WBC count, crystals, and Gram stain positivity were less helpful for diagnosis in this study, positive Gram stain and culture results from operative tissue specimens demonstrated greater effectiveness in diagnosing elbow NJSA.

## 1 Introduction

Native joint septic arthritis (NJSA) of the elbow is a relatively uncommon condition, with the prior literature describing rates of between 6 % and 9 % in prior retrospective studies (Weston et al., 1999; Kennedy et al., 2015; Maneiro et al., 2015). Several risk factors have been associated with elbow NJSA, including advancing age, rheumatoid arthritis, diabetes, skin ulcers, osteoarthritis, intravenous drug abuse, prior intra-articular corticosteroid injection, and previous joint prothesis (Mathews et al., 2010; Mehta et al., 2006; Weston et al., 1999). The consequences of elbow NJSA include a moderate loss of joint function and elevated mortality rate (Van Den Ende and Steinmann, 2012). Despite its clinical significance, limited literature exists regarding clinical presentation, management strategies, and overall outcomes of patients with elbow NJSA. This study aims to describe the contemporary experience with elbow NJSA at our institution over a 10-year period.

## 2 Materials and methods

A retrospective multicenter cohort study of adult patients (≥ 18 years) who were diagnosed with elbow NJSA and underwent surgical intervention at our institution's facilities during the period from January 2012 to December 2021 was undertaken. The diagnosis of septic arthritis was established based on clinical presentation, synovial fluid white blood cell (WBC) count, and the presence of positive bacterial culture obtained through aspiration or surgical intervention. Prior to the data collection, the study protocol received approval from the Institutional Review Board (IRB ID 22-005205). Furthermore, all participating patients granted their informed consent for the utilization of their medical records for research purposes under the Minnesota statute.

A comprehensive set of data were gathered and subjected to analysis, including demographics, clinical manifestations, underlying medical conditions, laboratory parameters, identified cultured organisms, medical treatment regimens, details of surgical procedures, recorded complications, and subsequent follow-up assessments. The Charlson comorbidity index (CCI) was calculated with age adjustment (Charlson et al., 1994). Data from patients who met the inclusion criteria were extracted to a pre-specified form and were collected and managed utilizing REDCap electronic data capture tools (Harris et al., 2019).

## 3 Results

During the study period, a total of 557 patients who met the inclusion criteria developed NJSA, with 19 patients (3.4 %) being diagnosed with elbow NJSA. Of the diagnosed patients, 14 (73.7 %) were male. The median age was 64 years (interquartile range, IQR: 45–75). The three most common comorbidities observed were diabetes mellitus, chronic kidney disease, and cerebrovascular disease, respectively. The median age-adjusted Charlson comorbidity index was 6 (IQR: 2–14), while the mean body mass index (BMI) was 31.1 kg m^−2^.

Among the patients, four (21.1 %) were immunocompromised, including two patients with hematologic malignancy, one patient with a myeloproliferative neoplasm, and one patient with an allogeneic hematopoietic stem cell transplant (HSCT). Additionally, three patients were on systemic steroids at the time of diagnosis (Table 1).

**Table 1:**
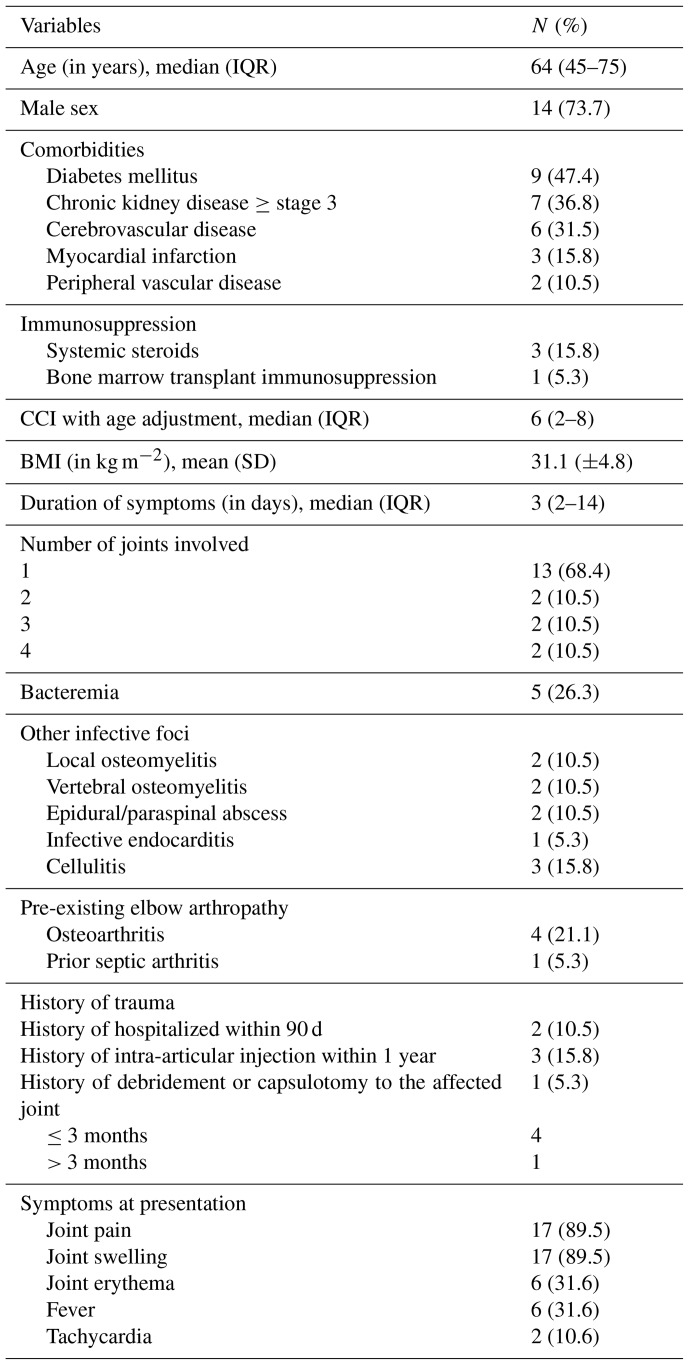
**Table 1**Baseline characteristics of 19 elbow native joint arthritis patients.

Data are *N* (%) unless otherwise specified. Abbreviations used in the table are as follows: BMI – body mass index; HSCT – hematologic stem cell transplant; IQR – interquartile range; CCI – Charlson comorbidity index; SD – standard deviation.

The duration of symptoms spanned a range of 1–618 d, with a median of 3 d (IQR: 2–14 d). Seven (36.8 %) patients presented within 48 h of symptom onset, five (26.3 %) presented between 2 and 7 d of onset, three patients (15.8 %) presented between 8 and 14 d of onset, and four (21.1 %) presented 14 d following onset. Joint pain and swelling were the predominant symptoms, reported by 17 patients (89.5 %). Further symptoms are shown in Table 1. Leukocytosis was observed on presentation, with a mean peripheral WBC count of 11 120 cells mm^−3^ (range: 2300–24 200 cells mm^−3^). The mean C-reactive protein (CRP) value was 114.70 mg L^−1^ (range: 5.09–558.10 mg L^−1^), and an erythrocyte sedimentation rate (ESR) was available for 17 patients and was elevated in 13 cases (76.5 %), with a mean of 63.91 mm h^−1^ and range of 5.0–253.4 mm h^−1^ (normal values: 2–20 mm h^−1^).

A total of 16 patients underwent joint aspiration, and their tissue specimens were sent from the operating room. The median synovial fluid WBC count was 43 139 cells mm^−3^ (IQR: 16 055–72 670 cells mm^−3^), characterized by a predominance of polymorphonuclear leukocytes (PMNs). Of these 16 patients, 13 (81.1 %) exhibited a polymorphonuclear leukocytes value greater than 85 %. Synovial fluid analysis revealed monosodium urate crystals in three patients (18.8 %). Positive Gram stain results were observed in 2 patients (12.5 %), while preoperative aspirate synovial fluid cultures were positive in 6 (37.5 %) of the 16 patients. Among the 16 patients with operative tissue samples, Gram stain and culture yielded positive results in 3 (18.8 %) and 9 (56.3 %) patients, respectively. The most prevalent pathogen identified in both specimen types was *Staphylococcus aureus*, with only one patient having methicillin-resistant *Staphylococcus aureus* (MRSA) and two patients having *Mycobacterium kansasii* (Table 2).

**Table 2:**
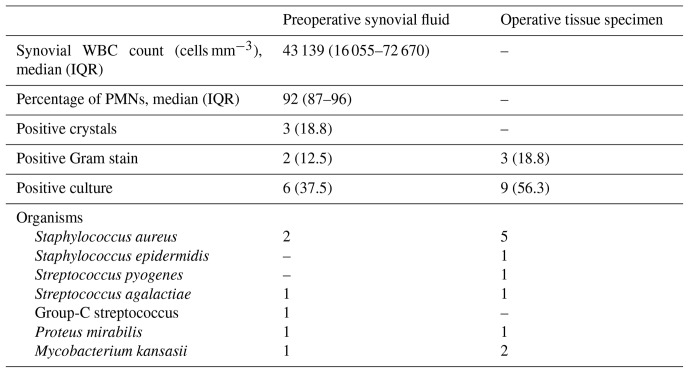
**Table 2**Characteristics of the synovial fluid and tissue joint specimen (*N*=16).

Data are *N* (%) unless otherwise specified. Abbreviations used in the table are as follows: IQR – interquartile range; PMNs – polymorphonuclear leukocytes; WBC – white blood cell.

A total of 18 patients underwent surgical intervention for joint drainage, with 13 (72.2 %) undergoing open debridement and 4 (22.2 %) undergoing open debridement and synovectomy (Table 3). Within 90 d, three patients (16.7 %) required repeat operations: one after arthroscopy and two after initial open debridement. All patients initially received broad-spectrum intravenous antibiotics, followed by targeted intravenous antibiotic treatment guided by culture results and antimicrobial susceptibility testing. Notably, vancomycin and piperacillin–tazobactam were the most frequently used empiric preoperative antibiotics, while ceftriaxone was predominantly utilized for definitive therapy. Intravenous antibiotics were administered for a median of 30 d (IQR: 22–44 d). Postoperatively, the most frequent complications observed were compromised range of motion and subsequent joint resections, which included total joint resection and joint arthroplasty.

**Table 3:**
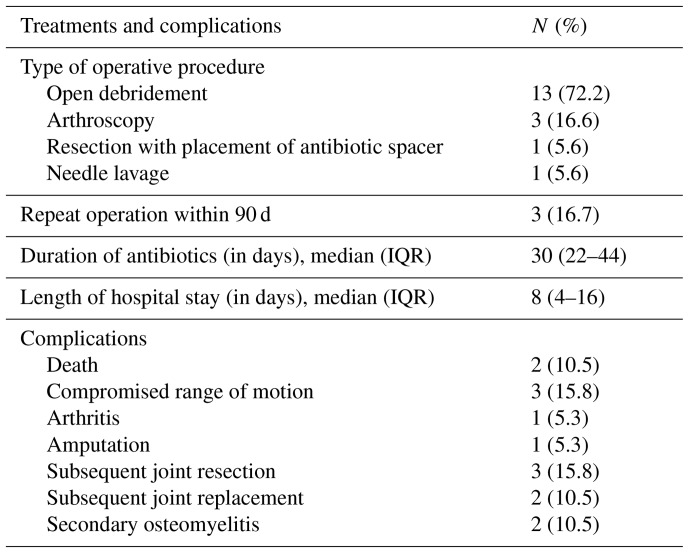
**Table 3**Treatments and outcomes.

Data are *N* (%) unless otherwise specified. IQR denotes interquartile range.

## 4 Discussion

Elbow NJSA occurs less frequently than NJSA of larger joints, such as hips and knees. In this review of cases of NJSA at our institution over a 10-year period, only 19 patients were diagnosed with elbow NJSA. The observed prevalence of 3.4 % was lower than that reported in previous studies from the UK (9.1 %) and Aotearoa / New Zealand (6.9 %) (Weston et al., 1999; Kennedy et al., 2015). The reduced prevalence of elbow septic arthritis compared with lower-extremity joints is likely multifactorial, potentially influenced by decreased mechanical stress and trauma in non-weight-bearing joints like the elbow. Furthermore, the smaller volume of synovial fluid in the elbow may limit bacterial proliferation and lower the risk of inoculum formation (Shirtliff and Mader, 2002). Consistent with a prior Mayo Clinic investigation, the median age of our patients was 64 years (Van Den Ende and Steinmann, 2012). Unlike some studies in which immunocompromised cases were linked to rheumatoid arthritis (Weston et al., 1999), we noted that immunocompromised patients were primarily associated with hematologic malignancy and stem cell transplantation.

Traditionally, a synovial fluid WBC count exceeding 50 000 cells mm^−3^ is considered a diagnostic criterion for acute septic arthritis (Shmerling et al., 1990; Sammer and Shin, 2009). Interestingly, only 37.5 % of aspirate cell counts in our study surpassed this threshold. Our IQR for the synovial fluid WBC count ranged from 16 055 to 72 670 cells mm^−3^. A study by Rashkoff et al. (1983) reported considerable variability in confirmed septic arthritis cases, ranging from 20 000 to 150 000 cells mm^−3^, further complicating definitive diagnosis in the absence of firm diagnostic criteria. Additionally, we noted the presence of monosodium urate crystals in 18.8 % of synovial fluid samples of elbow NJSA. Moreover, Hong et al. (2023) found that 21.3 % of NJSA cases presented with coexisting gout. Most cases diagnosed with septic arthritis concomitant with crystal presence in case reports involved the lower-extremity joints (Hong et al., 2023; Prior-Español et al., 2019; Weng et al., 2009). Importantly, the existence of crystals should not necessarily exclude the possibility of septic arthritis but rather be indicative of concomitant processes (Yu et al., 2003; Shah et al., 2007).

As observed in several other studies, *S. aureus* remained the most prevalent pathogen (Weston et al., 1999; Kennedy et al., 2015; Assunção et al., 2018; Moon et al., 2014; Maneiro et al., 2015; Van Den Ende and Steinmann, 2012; Mehta et al., 2006). While *S. aureus* prevalence in these studies varied from 56 % to 76 %, our study's positive culture rate for this pathogen was comparable at 56 %. The presence of negative cultures could be attributed to the of the timing of empiric antibiotic therapy, either in the emergency department or prior to hospitalization (Mehta et al., 2006).

The reoperation rate in our study resembled comparable studies, ranging from 9.09 % to 16.67 % for arthroscopic debridement (Moon et al., 2014; Van Den Ende and Steinmann, 2012). Notably, in our study, 2 of 13 (15.4 %) cases necessitated reoperation following open debridement. Consistent with observations in the knee joint, the reoperation rate associated with open debridement surpassed that of arthroscopic debridement. Specifically, reoperation rates ranged from 7.6 % to 71.43 % in the open-debridement group, compared with 6.3 % to 50.42 % in the arthroscopic-debridement group (Wirtz et al., 2001; Johns et al., 2017; Faour et al., 2019; Johnson et al., 2020). This discrepancy aligns with the hypothesis posited by Johns et al. (2017), who suggested that larger wounds incurred during open debridement may inflict greater damage to local tissues and facilitate the dissemination of contamination.

Contrasting with reported mortality rates as high as 30 %–50 % in other studies, our study recorded a mortality rate of 10.5 % (Weston et al., 1999; Van Den Ende and Steinmann, 2012). Open debridement was the most frequently utilized surgical approach in our cohort for joint drainage. Weston et al. (1999) found that open drainage was associated with decreased mortality in univariate analysis. Other prognostic factors that were consistently linked with poor outcomes included an age ≥ 60 years, pre-existing joint issues, an immunocompromised status, diabetes, multiple joint involvement, confusion, and *S. aureus* infection (Maneiro et al., 2015; Kennedy et al., 2015; Weston et al., 1999; Van Den Ende and Steinmann, 2012; Camp et al., 2017; Mehta et al., 2006).

We reported two male patients, aged 43 and 76, respectively, with *Mycobacterium kansasii* infections. One had a history of trauma, but neither was immunocompromised. Symptoms were indolent, lasting 1–2 years. Treatment involved open debridement; arthroscopy; and oral azithromycin, ethambutol, and rifampin for 274 and 372 d. Complications included osteomyelitis, repeated resection, and joint replacement in one case. Literature on *M. kansasii* infections in elbow septic joints is limited, but it typically involves cases with immunocompromised conditions such as the elderly, posttransplantation recipients, and those with acquired immune deficiency syndrome. Such cases commonly present with chronic joint pain and swelling and are managed with a combination of clarithromycin, ethambutol, rifampin, and surgical intervention (Okuno et al., 2020; Sugiyama et al., 2020; Friedman and Ike, 1993).

Limitations of this study include its retrospective nature as well as the relatively small subset of patients affected with NJSA over the study period, restricting the comprehensive study of outcome-associated factors. Subsequent meta-analyses integrating data from a series of cases could improve understanding of elbow septic arthritis. Nevertheless, it is noteworthy that our study holds significance as one of the only investigations solely dedicated to elbow septic arthritis.

## 5 Conclusions

Native septic joint arthritis of the elbow is less common compared with other joints. The prevalence of patients in our study population with both low WBC counts and crystals in the synovial fluid, which can be misleading for the diagnosis of crystal arthropathy, was found to be 3.4 %; thus, this warrants careful consideration during clinical evaluation. Concomitant conditions, such as crystallin arthropathy and infection, should always be investigated. Our observations also highlight the emergence of atypical *M. kansasii* infections. Despite our lower mortality rate compared with some reports, the outcomes of elbow NJSA remain serious, particularly due to the high rate of reoperation, reinforcing the importance of early diagnosis, prompt initiation of appropriate treatment, and cautious postoperative care.

## Data Availability

The datasets generated during and/or analyzed during the current study are available from the corresponding author upon reasonable request.
